# Identification of Chinese dietary patterns and their relationships with health outcomes: a systematic review and meta-analysis

**DOI:** 10.1017/S1368980024001927

**Published:** 2024-10-14

**Authors:** Xue Feng Hu, Rui Zhang, Hing Man Chan

**Affiliations:** Chemical and Environmental Toxicology Program, Department of Biology, University of Ottawa, Ottawa, ON K1N 6N5, Canada

**Keywords:** Chinese diets, Dietary pattern, CVD, Cancer, Metabolic syndrome, Systematic review and meta-analysis

## Abstract

**Objective:**

China has been undergoing a rapid nutrition transition in the past few decades. This review aims to characterise commonly reported dietary patterns in Chinese populations and their associations with health outcomes.

**Design:**

We searched PubMed, Embase and CNKI from inception to June 2020 to identify observational studies reporting dietary patterns or the associations between dietary patterns and health outcomes. Information regarding dietary patterns, their association with health outcomes and other related items was collected.

**Setting:**

Chinese population and Chinese immigrants.

**Participants:**

Not applicable.

**Results:**

Results from 130 studies with over 900 000 participants were included. Six dietary patterns were identified: traditional whole-grain diet (Traditional WG), traditional non-whole-grain diet (Traditional NWG), plant-based diet (Plant-based), animal food diet (Animal-food), Western energy-dense diet (Western) and other unclassified diets (Unclassified). The Plant-based diet was associated with a reduced risk of CVD and cancer from prospective studies, reduced risk of diabetes, hypertension, cognitive impairment and depressive symptoms from all study designs. The Traditional WG diet was associated with a reduced risk of diabetes and hypertension. Animal-food diet is associated with a range of metabolic diseases, and Western diet was associated with increased risks of obesity and depressive symptoms.

**Conclusion:**

Multiple dietary patterns identified reflect the diversity and transitioning of the Chinese diet. A healthy Chinese diet, comprising both the Traditional WG and Plant-based diets, was associated with reduced risks of specific undesirable health outcomes. Promoting this healthy diet will improve public health among the Chinese populations.

The Chinese diet has evolved over thousands of years, influenced by the country’s vast geography, numerous ethnic groups and rich cultural history. The traditional Chinese diet comprises cereals and vegetables with few animal foods and is considered to be healthful when adequate intake levels are achieved^(1,2)^. Since the 1950s, China has been undergoing a nutrition transition towards a more westernised diet in parallel with rapid socio-economic and demographic changes^(3–5)^. This transition has helped overcome food scarcity and improve the nutritional status of the Chinese population over the past few decades^(5,6)^, but it has also led to unhealthy outcomes^(7,8)^. Evidence from the Cornell China Study, one of the most comprehensive nutrition studies conducted in the 1980s^(9)^, shows that counties in China with higher animal-based food consumption were more likely to have had higher death rates from Western diseases than those with higher plant-based food consumption^(1)^. The burden of chronic diseases has been increasing rapidly in China. From 1990 to 2010, the age-standardised mortality of diabetes mellitus and ischaemic heart disease increased by 52·3 % and 31·6 %, respectively^(7,10)^. It was estimated that dietary risk factors (mainly diets low in fruits, vegetables, whole grains, and nuts and high in Na) accounted for 16·3 % of this increase^(7,10)^. Thus, an update on the characterisation of the Chinese diet to define beneficial and detrimental components is necessary for policymakers and public health professionals to provide up-to-date dietary advice to revert undesirable dietary transitions in developing countries similar to that in China^(11–14)^.

In nutritional epidemiology, it is often preferable to estimate adherence to a certain dietary pattern (e.g. the Mediterranean diet) than to analyse the individual dietary components in relation to the population’s health status^(15)^. This is because people do not consume isolated nutrients or single foods, and different nutrients and foods interact with each other^(15)^. Therefore, dietary pattern analysis can provide more integrated dietary information to explore the associations with health outcomes.

China has a population of 1·4 billion as of 2018^(16)^, and it is estimated that over 45 million people of Chinese ethnicity live in Southeast Asia, North America, Europe, Australia, Japan, South Korea and other countries^(17)^. Chinese cuisine is highly diverse and is strongly related to geographic differences in agricultural practices and food availability, as well as ethnic and other cultural and socio-economic factors^(18–23)^. Therefore, identifying an ‘overall’ or ‘unique’ dietary pattern for the Chinese population is neither feasible nor of particular interest. Instead, identifying common dietary patterns and summarising their key features would better characterise the Chinese diet. Many studies have reported on Chinese dietary patterns and their relationship with health outcomes in the last two decades. However, a systematic review and appraisal of published Chinese dietary pattern studies is lacking.

In this study, we performed a systematic review of the literature to characterise the dietary patterns among Chinese populations. In addition, we assessed the associations between the identified Chinese dietary patterns and health outcomes to characterise healthy Chinese dietary patterns.

## Methods

### Searches

We used three databases, Ovid Medline (https://ovidsp.ovid.com/), Embase (https://www.embase.com/home) and China National Knowledge Infrastructure (CNKI, https://cnki.net/), to find all published studies that used modelling techniques to identify dietary patterns in the Chinese population. Search terms included combinations of ‘diet*’ AND ‘pattern*’ AND (‘China’ OR ‘Chinese’) with specific terms including ‘food’, ‘intake’, ‘principal component*’, ‘PCA’, ‘factor*’, ‘FA’ and ‘cluster*’ for Medline and Embase. Similar terms in Chinese were used for CNKI. The databases were searched from inception until June 2020, and no language restrictions were set. The reference lists of identified studies and other literature sources, including Cochrane Database of Systematic Reviews, Google Scholar, were also searched.

### Study inclusion and exclusion criteria

Studies that fulfilled the following *a priori* eligibility criteria were included: (a) original study, (b) used a FFQ or 24-h dietary recall, (c) Chinese population and (d) published in English or Chinese. The exclusion criteria were (a) non-original report, (b) study of individual foods or nutrients, (c) no dietary pattern reported and (d) study only of infant feeding. If more than one paper was published from the same study with identical dietary patterns and health outcomes, only the most recent paper was included.

### Study selection

**Fig. 1:**
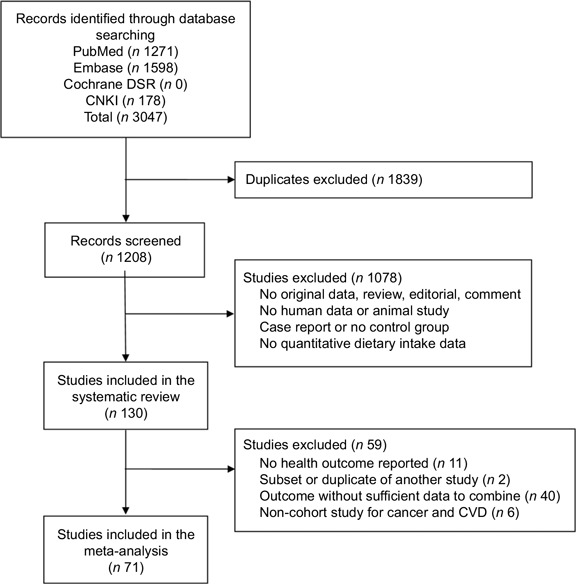
Flow chart describing the study selection process

The initial literature search yielded 3047 records, of which 1839 duplicates were excluded. Of the remaining 1208 records, 1079 were excluded for the following reasons: unrelated reports or reviews; no dietary pattern reported; no healthy participants included; study of diets in non-human species; and no access to the full text. A total of 130 studies were included in the systematic review to summarise the major dietary patterns in the Chinese population^(24–153)^. Another fifty-nine studies were excluded based on the following exclusion criteria: no health outcome reported (*n* 11); subset or duplicate of another study (*n* 2); health outcome without sufficient data (fewer than three studies for each outcome) (*n* 40); and non-cohort study for cancer and cardiovascular health outcomes (*n* 6). Ultimately, seventy-one studies were included in the meta-analysis to investigate the associations between identified dietary patterns and health outcomes (Fig. 1).

### Data extraction

X.F.H. conducted the search using the databases listed above. The data were initially extracted by R.Z. and were 100 % validated independently by another two team members (Y.Y.L. and C.Y.C.). Two databases were compiled separately for the review. One database contained the relevant characteristics of the included studies: authors, publication year, journal title, geographical area, years data were collected, study name and design if available, specific populations studied, sample size, dietary intake assessment methods, statistical methods, food groups or items used, reported dietary pattern names, percentage of the total variance of original food items explained by the dietary patterns (for studies using factor analysis or principal component analysis), statistical adjustments made in the analysis and any other relevant information about the study. The other database consisted of effect size measures between identified dietary patterns and health outcomes (OR, hazard ratio, relative risk (RR) or prevalence ratio) and their standard errors from the published data. Continuous outcomes (e.g. systolic blood pressure) were not included in the data extraction and synthesis.

### Quality assessment of included studies

For studies reporting the association between dietary patterns and health outcomes, we assessed their quality with a modified nine-point version of the Newcastle–Ottawa scale (see online supplementary material, Supplemental Material 2), focusing on dietary assessment, dietary pattern identification and confounding control^(154)^. Study quality was categorised as high (score ≥ 7), medium (score 5–6) or low (score ≤ 4).

### Definition of dietary pattern categories

The classification of dietary patterns varied among the studies. Common methods included classification based on typical food groups (e.g. vegetables/fruits, meat, etc.)^(29,34,83)^, time period or dietary transition (e.g. traditional, Western or modern)^(53,110,121,133)^, associations with health (e.g. healthy)^(89,93)^ or other characteristics (e.g. balanced, prudent, high protein, macho, condiment or beverage)^(47,56,71,96)^. To better summarise the identified dietary patterns, we grouped them into the following six categories: traditional whole-grain diet (Traditional WG, with wheat and other cereals as staple foods), traditional non-whole-grain diet (Traditional NWG, with rice as the staple food), plant-based diet (Plant-based), animal food diet (Animal-food), Western energy-dense diet (Western) and unclassified diets (Unclassified, including all other diets). The re-classification of dietary patterns was kept minimal and only when deemed essential to facilitate the comparison and interpretation of results from different studies. The main re-classification divided the ‘traditional’ dietary pattern into either whole-grain or non-whole-grain, depending on whether relevant food items/groups (e.g. whole grains, coarse grains and other cereals) were reported as high factor-loading items for that dietary pattern. No dietary pattern was re-classified to either traditional pattern if the term was not used in the original study. A similar principle was applied to other dietary patterns. An additional ‘fruit-rich diet’ was named for one cohort study^(29)^, which reported two plant-based dietary patterns (fruit-rich *v*. vegetable-rich) showing opposite associations with multiple health outcomes. The re-classification was performed independently by three team members (R.Z., Y.Y.L. and C.Y.C.), and any discrepancies were resolved by consensus or consulting with senior members (X.F.H. or H.M.C.). Dietary patterns were re-classified before the meta-analysis to avoid potential bias in analysing the associations between dietary patterns and health outcomes. The names of the original dietary patterns and the re-classified patterns for each study are presented in online supplementary material, Supplemental Material 1.

### Data analysis

The association between a given dietary pattern and health outcome was analysed using an inverse-variance weighted random-effects model. Most of the included studies (61 out of 71) used factor analysis, principal component analysis or reduced rank regression to identify dietary patterns and reported their results in tertiles, quartiles or quintiles of dietary pattern scores (with intermediate categories omitted in some studies). These studies estimated the effect size by comparing the group with the highest dietary pattern scores (Q3, Q4 or Q5) to the group with the lowest dietary pattern scores (Q1). The remaining ten studies identified dietary patterns using cluster analysis, comparing the group with a dietary pattern of interest (e.g. Western) to the group with another dietary pattern (e.g. Traditional). Results from both types of studies were pooled together. Heterogeneity was quantified with the *I* ^*2*^ statistic^(155)^. The relative influence of each study on the pooled estimates was determined by omitting one study at a time. Four sets of sensitivity analyses were conducted to investigate factors that may bias the associations between identified dietary patterns and health outcomes: (1) the dietary pattern identification method (studies using factor analysis or principle component analysis *v*. all studies), (2) study design (cohort studies *v*. cross-sectional studies), (3) participants’ age of included studies (studies with adult participants only *v*. all studies) and (4) combining the Plant-based diet and Traditional WG diet into a Chinese healthy diet (only Plant-based diet was included if both dietary patterns were reported in the same study).

The following health outcomes were summarised: CVD from cohort studies, including fatal and non-fatal stroke, acute myocardial infarction, and CHD; cancer from cohort studies, including fatal and non-fatal events; metabolic outcomes from all study designs, including type 2 diabetes, hypertension, general obesity (defined using BMI), abdominal obesity (defined using waist circumstance), cognitive impairment and depressive symptoms. Lipid disorders and gestational diabetes were reported as well. However, no synthesis was done due to an insufficient number of studies. Additional information about the Population, Intervention, Comparison, Outcomes and Study design criteria is available in Table S1 (see online supplementary material, Supplemental material 2).

The review protocol was registered in the Prospero database under the registration number: CRD42022321001. This review was conducted as per PRISMA 2020 guidelines (http://www.prisma-statement.org/PRISMAStatement).

## Results

### Study characteristics

The 130 studies included in the systematic review collected data from the Chinese population living in mainland China (*n* 19 for national or multiregional studies, *n* 83 for studies conducted in a single province or city), Hong Kong Special Administrative Region (SAR) (*n* 13), Macao SAR (*n* 1), Taiwan (*n* 4) and Singapore (*n* 7), as well as Chinese immigrants living in the USA and Malaysia (*n* 3) (Table 1, Fig. S1). Nine studies reported dietary data collected before 2000, sixty-three studies reported data collected between 2001 and 2010, and the remaining fifty-eight studies collected dietary data after 2011. One-third (*n* 43) of the included studies used a cohort design, 58 % (*n* 76) were cross-sectional studies and the rest were case–control studies (*n* 11). About 85 % of the studies (*n* 110) used an FFQ to assess dietary intake. Factor analysis or principal component analysis (*n* 108) were the most common methods used to derive dietary patterns. The longitudinal associations between dietary patterns and CVD were examined in six studies, and those with cancer were examined in another five. The most commonly investigated health outcomes were metabolic conditions, that is, diabetes (*n* 15), abdominal obesity (*n* 14), hypertension (*n* 14) and general obesity (*n* 9). An increasing number of studies reported cognitive and mental health outcomes after 2010 and gestational outcomes after 2015. Dietary patterns were identified using food groups in sixty-three studies and food items in the remaining studies (see online supplementary material, Supplemental Material 1). Researchers included 11–55 food groups and 33–280 food items. Ten studies focused on children (< 18 years old), seventeen studies reported on the elderly only (≥ 60 years old or average age over 60 years), and the remaining studies were conducted in adults. The sample size of included studies ranged from 130 to 481 242, and the total number of subjects in the included studies was 902 620. Results of the participants were included in multiple analyses of associations with health outcomes, with a total number of 2 076 893.

**Table 1:** Characteristics of the studies included in the systematic review

Characteristic	Study count
Region	
National and multiregional	19
Hong Kong SAR	13
Macao SAR	1
Taiwan	4
Single province in mainland China	83
Singapore	7
Chinese immigrants living in other countries	3
Study design	
Cohort	43
Cross-sectional	76
Case–control	11
Publication language	
English	114
Chinese	16
Dietary intake assessment method	
FFQ	110
24-h recall^*^	20
Dietary pattern derivation method	
Factor analysis (principal component analysis)	108
Cluster analysis^†^	14
Reduced-rank regression	7
Structure equation	1
Dietary data collection period^‡^	
Before 2000	9
2001–2010	63
After 2011	58
Average dietary pattern identified	3
Health outcome included in meta-analysis^§^	
CVD	6
Cancer	5
Diabetes	15
Hypertension	14
General obesity	9
Abdominal obesity	14
Depressive symptoms	5
Cognitive impairment	6

SAR, Special Administrative Region.

* Four studies reported both FFQ and 24-h recall.

† Ten studies used factor analysis and cluster analysis together.

‡ Time of completion for multi-wave cohort studies.

§ Some studies reported multiple health outcomes.

Most studies provided adequate information on the selection of the study population, the methods used to assess dietary intake and derive dietary patterns, the ascertainment of health outcomes, and the statistical methods used to analyse the associations between dietary patterns and health outcomes. In total, 117 studies were assessed for quality, as thirteen studies only reported dietary patterns and did not investigate their relationship with health outcomes. Overall, 81·2 % of all studies assessed (95 out of 117) and 86·1 % of the studies included in the meta-analysis (62 out of 71) were rated as high quality. Papers published in Chinese were generally more concise, with fewer tables and supplementary materials. However, the methods used to assess dietary intake, derive dietary patterns and adjust for covariates were similar to those of the studies published in English.

### Summary of dietary patterns

Of the seventy-nine studies reporting traditional Chinese dietary patterns, forty-six reported Traditional WG (featuring whole grains, wheat, fresh vegetables and fruits, legumes, soyabean and products), fifty-four reported Traditional NWG (featuring rice, fresh vegetables and pork) and twenty reported both (Tables 2 and 3). In addition, seventy-seven studies reported Plant-based (featuring fresh vegetables and fruits, legumes, soyabean and products, and mushrooms and fungi), eighty-three reported Animal-food (featuring red meat and processed meat) and eighty-eight reported Western (featuring soft drinks and snacks) diets. Finally, forty-six reported Unclassified diets, among which high-salt condiments and alcohol were the two most commonly reported groups.

**Table 2:** Classified dietary patterns and most frequently reported food groups or items

Dietary pattern	No. of studies	Food groups or items most frequently reported
Traditional whole-grain	46	Whole grains, coarse grains, wheat, other cereals, fresh vegetables, fresh fruits, legumes, soyabean and products, fish and seafood, tubers, nuts, tea
Traditional non-whole-grain	54	Rice, fresh vegetables, pork, poultry, fish
Plant-based	77	Fresh vegetables (dark green and leafy), fruits, legumes, soyabean and products, mushrooms and fungi, whole grains, coarse cereals, nuts, fish and seafood
Animal-food	83	Red meat (pork, beef and lamb), processed meat, poultry, organ meat, fish and seafood, egg, fat/oil
Western	88	Soft drink, juice, fast food, French fries/chips, milk and dairy products, cakes, snacks, desserts, processed products, alcohol
Unclassified^*^	46	Condiments, alcohol, and combinations of food groups or items that do not fit into the other defined patterns

* High-salt pattern (i.e. condiment pattern) and beverage pattern were the two main unclassified patterns.

**Table 3:** Number of studies reporting the classified dietary patterns

	Traditional whole-grain	Traditional non-whole-grain	Plant-based	Animal-food	Western	Unclassified
Traditional whole-grain		20	13	20	35	20
Traditional non-whole-grain			23	25	38	23
Plant-based				62	47	13
Animal-food					30	20
Western						35
Unclassified						

One to six dietary patterns were reported per study, with an average of three. Plant-based and Animal-food diets were the most frequently reported in the same study (*n* 62), followed by Plant-based and Western (*n* 47). Traditional WG and Plant-based diets had similar lists of high factor-loading food groups or items and were the least likely to be identified in the same study (*n* 13). Among regions, studies conducted in Hong Kong SAR, Singapore and coastal regions like Shanghai and Tianjin were more likely to report Plant-based and Animal-food diets. In national and multiregional studies, traditional Chinese dietary patterns were often further categorised into traditional northern (wheat-based) and traditional southern (rice-based) diets. No clear temporal trend was observed in the reported dietary patterns. However, studies before 2015 were more likely to focus on diseases such as cancer, CVD and metabolic conditions. Recently, studies started to focus on mental health conditions and certain sub-populations, for example pregnant women.

### Dietary patterns and health outcomes

**Fig. 2:**
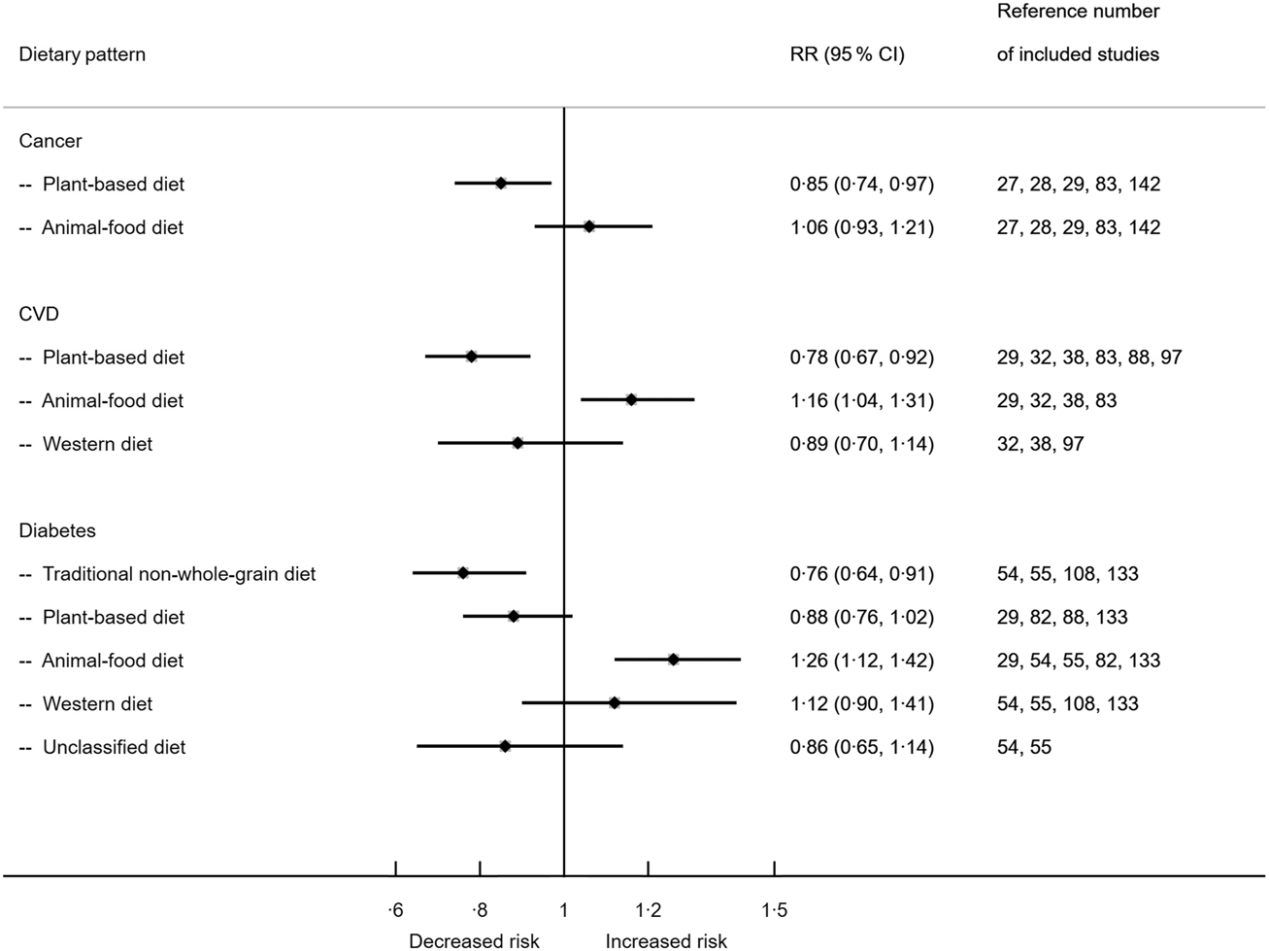
Associations between dietary patterns in Chinese population and CVD, cancer and diabetes from cohort studies. RR, relative risk.

Figure 2 shows the associations between dietary patterns, CVD, cancer and diabetes in cohort studies. The RR associated with the Plant-based diet was 0·85 (95 % CI 0·74, 0·97) for cancer and 0·78 (95 % CI 0·67, 0·92) for CVD. The Animal-food diet was associated with an increased risk of CVD (RR 1·16, 95 % CI 1·04, 1·31) and diabetes (RR 1·26, 95 % CI 1·12, 1·42). The Traditional NWG diet was associated with a decreased risk of diabetes with RR 0·76 (95 % CI 0·64, 0·91).

**Fig. 3:**
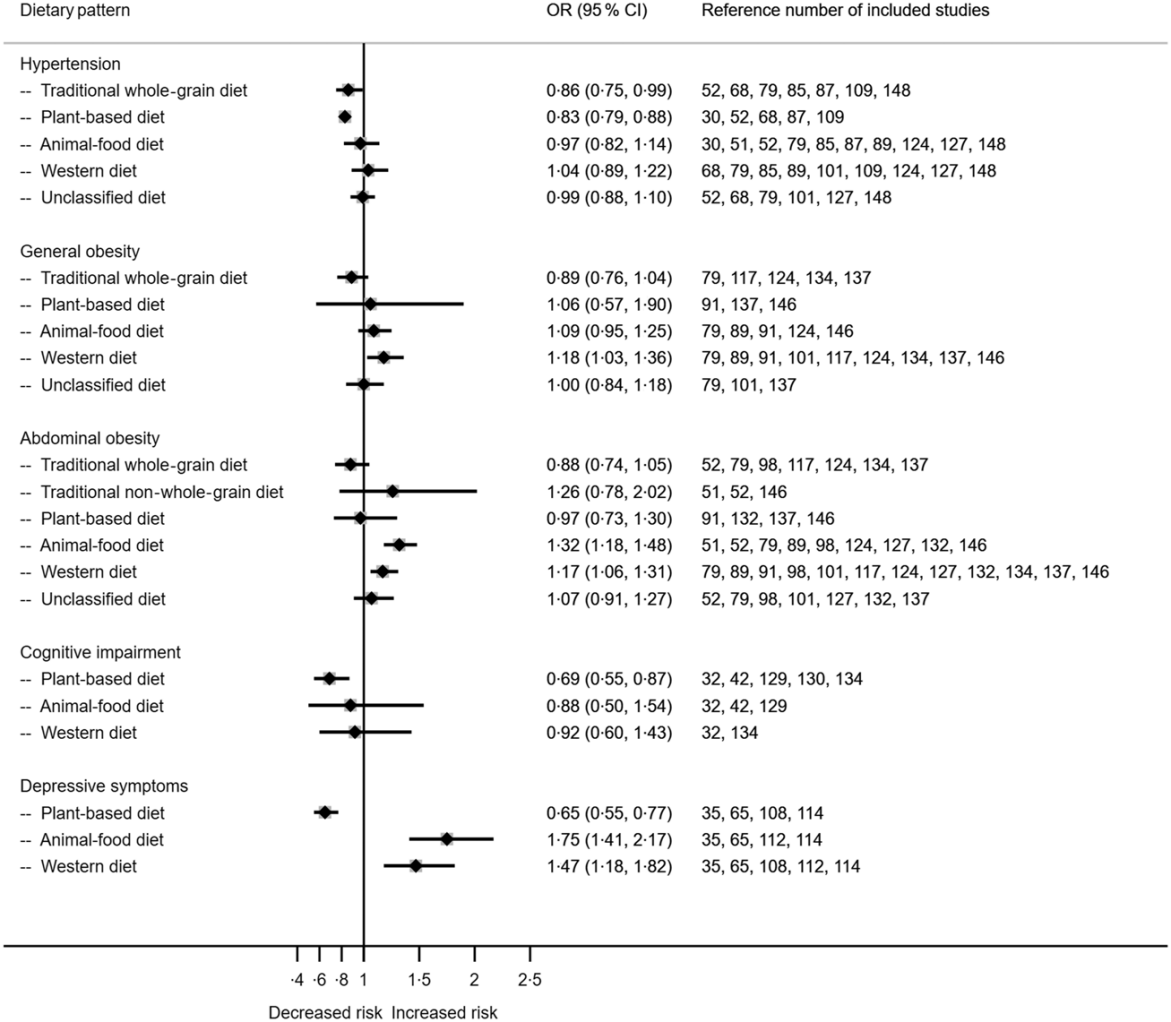
Associations between dietary patterns in the Chinese population and hypertension, general obesity, abdominal obesity, cognitive impairment and depressive symptoms.

Figure 3 shows the associations between dietary patterns, selected metabolic and other health conditions from studies with both cohort and cross-sectional designs. Greater adherence to the Traditional WG diet resulted in decreased risk of hypertension (OR 0·86, 95 % CI 0·75, 0·99). Animal-food diet was associated with an increased risk of abdominal obesity (1·32, 95 % CI 1·18, 1·48). Meanwhile, the Western diet was associated with an increased risk of general obesity (OR 1·18, 95 % CI 1·03, 1·36) and abdominal obesity (OR 1·17, 95 % CI 1·06, 1·31). Adherence to the Plant-based diet was associated with a reduced risk of cognitive impairment (OR 0·69, 95 % CI 0·55, 0·87) and depressive symptoms (OR 0·65, 95 % CI 0·55, 0·77). Animal-food and Western diets were associated with increased risks of depressive symptoms (OR 1·75, 95 % CI 1·41, 2·17 and OR 1·47, 95 % CI 1·18, 1·82, respectively). Forest plots with more details for each outcome are provided in online supplementary material, Supplemental Fig. 6–13.

Sensitivity analysis showed that the pooled estimates between dietary patterns and health outcomes were similar with or without studies using cluster analysis (see online supplementary material, Supplemental Fig. 2), with or without cross-sectional studies (see online supplementary material, Supplemental Fig. S3, for diabetes only), with or without participants aged 18 years or less (see online supplementary material, Supplemental Fig. 4), and with and without Plant-based diet and Traditional WG diet combined into one (see online supplementary material, Supplemental Fig. 5).

## Discussion

This is the first systematic review and meta-analysis of Chinese dietary pattern studies, covering data collected from more than 900 000 participants living in mainland China, Hong Kong SAR, Macao SAR, Taiwan and Singapore, as well as Chinese immigrants living in the USA and Malaysia. The results of the systematic review have identified six commonly reported dietary patterns, including: (1) the Traditional WG diet (with wheat and other cereals as staple foods), (2) Traditional NWG diet (with rice as the staple food), (3) plant-based diet (Plant-based), (4) animal food diet (Animal-food), (5) Western energy-dense diet (Western) and (6) unclassified diets (Unclassified, including all other diets). Among these, a ‘healthy Chinese diet’ characterised by traditional style, whole grain and plant-based components was consistently associated with decreased risk of various health outcomes. In the rest of the discussion, we would like to focus on the following two perspectives: the diversity of Chinese diets and their explanation, the features of a healthy Chinese diet and how it compares to other healthy diets.

China has experienced a rapid diet transition over the last few decades^(6,156)^. The diet transition occurred in two ways. In one way, the diet transition in China towards a westernised diet is unfavourable. From 1952 to 1992, cereal consumption increased from 540 g/d to 645 g/d, while the percentage of coarse grains decreased from 70 % to 16 %^(6)^. Data from the CHNS showed that the average daily intake of whole grains further decreased by approximately 10 % from 1997 to 2011^(157)^. Meanwhile, the consumption of animal-sourced foods, half of which were pork and pork products, tripled from 30 g/d to 103 g/d^(6)^. While the total energy intake decreased, the percentage of energy intake from fat tripled from 7·6 % to 22·5 % and further increased to 27·3 % in 1997 and 35·6 % in 2015^(24,79,110,158)^. Our results also reflect this diet transition. Two-thirds of the included studies reported either the Animal-food or the Western diet, and fifty-three reported both. Recent data have shown that the transition toward the Animal-food and Western diets is ongoing. For example, the average daily total meat consumption in China increased from 75 g to 90 g (normalised to a 2000 kcal diet) between 2002 and 2012^(159–161)^. Statistics from the FAO’s Food Balance sheet also suggested that the increase in meat consumption continued after 2012^(8)^. In the present review, Animal-food diet was found to be associated with increased risk of CVD and diabetes from cohort studies. The Western diet and Animal-food diet were found to be associated with increasing risk of obesity and depression. These are in agreement with the literature^(162,163)^. The other associations, for example, between Animal-food diet and cancer and between Western diet and diabetes, are also positive; however, they are insignificant. If the transition from a plant-based diet to animal food and the Western diet in China cannot be controlled or reverted, the burden of chronic diseases in China, especially those attributed to poor diet quality, will continually increase. The other way of transitioning towards a more balanced and diverse diet is hard to capture in the current review as many of the studies included are cross-sectional. Fresh fruit and vegetables are becoming more available and affordable for certain geographical and rural areas in China due to improved supply chains, etc^(22,164)^. Evidence suggests that vegetable consumption declined consistently until 1992, stabilised and then increased thereafter^(3,157)^. As positive changes in the Chinese diet, fruit consumption has been increasing over time^(4)^. In contrast, the average Na intake has been decreasing, although it remains above the WHO’s recommendation^(165)^.

As expected, various dietary patterns were reported in the included studies. This could be explained mainly by three factors: (1) geographic variations in agriculture practices and food availability, (2) heterogeneity in social and economic status, cultural background of included participants, dietary pattern naming conversion between research groups and (3) diet transition among Chinese population for the last few decades. China is a large and diverse country, both geographically and culturally^(166)^. For example, wheat and other cereals are staple foods in northern China, whereas rice is a staple food in southern China^(23)^. Moreover, residents of coastal regions and southeastern China have greater access to fish and seafood than those of interior regions^(52,53)^. In addition, certain vegetables are frequently consumed in some regions but rarely in others. More than half of the included studies were conducted in a single province or sub-administrative region. The food lists used to assess dietary intake and the identified dietary patterns varied across these studies. Differences in socio-economic development level (e.g. western *v*. eastern China; rural *v*. urban) are another important factor determining the variability and affordability of nutritious food^(22)^. Such differences were more likely to be captured by studies using the two national nutrition surveys, the Chinese National Nutrition and Health Survey (CNNHS) and the Chinese Health and Nutrition Survey (CHNS)^(167–169)^. Cultural tradition, ethnic background, dietary habits, food preparation and cooking methods play influential roles in people’s dietary choices^(18–20,23)^. Besides the above-mentioned factors, the naming and reporting of dietary patterns could be arbitrary. The same dietary pattern could be named ‘healthy’ based on its relationship with health outcomes, ‘vegetable-rich’ based on food groups or ‘traditional’ due to cultural preference.

Despite rapid dietary changes, traditional diets remained prominent. Two-thirds of the included studies reported at least one traditional dietary pattern. In this review, we re-classified them into either Traditional WG or Traditional NWG, depending on whether food groups or items related to whole grains are a high-loading factor during the dietary pattern identification process. Given the sharp decline in whole grain consumption in the Chinese population, it is reasonable to assume that participants who adhere to the Traditional WG diet consume more whole-grain foods than those adopting other diets. However, this does not necessarily mean they consume a ‘whole-grain diet’. Besides the difference in whole grains, wheat is more likely to be a high factor-loading item (staple food) in the Traditional WG diet and rice in the Traditional NWG identified in this review. The traditional WG diet is more likely to be associated with a lower risk of metabolic risk factors (see online supplementary material, Supplemental Fig. 8–11). However, it is out of the scope of this review to compare the health impact of wheat-based diet and rice-based diet among the Chinese population, given many factors may bias such comparison. There has been concern about rice consumption and the risk of diabetes among the Chinese population^(170)^. The present review found inconclusive evidence of this relationship. Traditional NWG diet, more likely with rice as a staple food, showed a negative association with diabetes (Fig. 2, see online supplementary material, Supplemental Fig. 8). Many of the studies reporting the Traditional NWG diet were conducted in wealthy coastal provinces in southern China, where dietary quality, social development and healthcare system are different from the other regions. Second, evidence suggests that rice cultured in southern China has higher amylose contents and a lower glycemic index^(171)^. It may not be feasible to define a standard ‘traditional Chinese diet’. However, healthy Chinese diets may share common features.

The Traditional WG diet shared more similarities with the Plant-based diet than the Traditional NWG diet in terms of high factor-loading food groups and their associations with health outcomes. Moreover, few studies (13 out of 130) reported both Traditional WG and Plant-based diets (Table 3). The shared food groups/items between the two diets are: whole grains (coarse cereals), fresh fruits and vegetables, soyabeans and products, mushrooms and fungi, nuts, and fish and seafood. Moreover, the sensitivity analysis revealed that the associations of these combined diets and many health outcomes, especially metabolic conditions, followed the same trends with similar magnitudes as those for each of the patterns individually (e.g. effect size 0·86 *v*. 0·83 for hypertension, 0·78 *v*. 0·79 for diabetes; see online supplementary material, Supplemental Fig. 5). Therefore, these two diets appear to be essentially the same dietary pattern reported under different names in the literature. This combined ‘healthy Chinese diet’ can be broadly characterised by an abundance of plant foods, a preference for whole grains over refined grains, the inclusion of soyabeans and products, mushrooms, and fungi as integral components of the daily diet, and a balanced diet (e.g. not a vegetarian diet) with a high proportion of fish and seafood.

The identified healthy Chinese diet shares features similar to other well-known healthy diets, such as the Mediterranean^(172,173)^ and Japanese diets^(174)^. The common food groups or items in the healthy Chinese diet are also key elements in established dietary indexes, such as the Mediterranean Diet Score^(175)^, Healthy Eating Index^(176)^, Diet Quality Index^(177)^ and DASH score^(178)^. The associations between this healthy Chinese diet and health outcomes were comparable to other dietary patterns. For example, adherence to the healthy Chinese diet was associated with a 22 % decrease in CVD incidence or mortality, within the range of 12 % to 31 % from other reviews^(179–182)^. This dietary pattern was also associated with a 15 % decrease in cancer incidence and mortality (mainly from colorectal cancer and breast cancer), in agreement with the latest evidence on diet and cancer^(182,183)^. The evidence for the negative associations between the healthy Chinese diet and risk of CVD, cancer and diabetes is strong as they are based on results from cohort studies. Moreover, the association between the healthy Chinese diet or its components and metabolic syndrome was in accordance with reviews on other healthy diets^(179,184–187)^. Finally, even the evidence for the negative associations between the healthy Chinese diet and depressive symptoms and cognitive impairment is only moderate as they were only based on mixed cohort studies and cross-sectional studies; the negative association is similar to that reported for the Mediterranean diet^(188)^.

This study holds scientific, clinical and public health implications. First, the findings of the present review shed light on additional exploration into the health-promoting components of the typical traditional Chinese diet. Second, the clinical efficacy of dietary interventions characterised by a healthy Chinese dietary style merits further investigation. Third, these findings provide scientific evidence informing public health nutrition policies to address the escalating burden of chronic diseases in China and elsewhere where Chinese diets are common.

Our study has several limitations. Some are related to the scope of the review and dietary assessment in general. The review included dietary pattern studies only. Studies focus on the beneficial effects of a single food item or group, key nutrients, or certain types of meals that were not within the scope. Thus, certain healthy components of the Chinese diet, such as green tea, mushrooms and tofu, might have been overlooked. Furthermore, the standard dietary assessment method may not capture certain diet features. For example, food preparation and cooking methods, timing of meals, and the use of certain herbs and spices. This may have little impact on summarising the existing Chinese dietary patterns; however, it may compromise our effort to characterise a healthy Chinese diet and its components. Some are related to the heterogeneity of included studies. For example, (1) diets were assessed using different tools (e.g. FFQ *v*. 24-h recall) to different degrees of detail (e.g. number of food groups or items analysed), (2) dietary patterns were retained with different eigenvalue and factor-loading cut-offs, and named with different conventions, (3) study design, health outcome definition, statistical analyses and covariate adjustments varied among studies as well. We conducted various sensitivity analyses, showing that the associations observed between dietary patterns and health outcomes are robust regardless of the above-mentioned factors. Some are related to our data synthesis. The renaming of dietary patterns, including cross-sectional studies and case–control studies for certain health outcomes, and summarising studies from different geographic levels, regions and social development statuses may also introduce additional heterogeneity to the meta-analysis. The heterogeneity resulted in the relatively modest effect size observed in the associations between dietary patterns and health outcomes. Nonetheless, it is clear that the Traditional WG and Plant-based diets are ‘healthier’ than the Animal-food and Western diets.

Further work needs to be done to understand Chinese dietary patterns and healthy Chinese diets. First, it is worth exploring if any unique healthy food item or group is being ignored by current dietary pattern analysis. Second, although healthy dietary patterns share similar characteristics, one pattern may not work for all the Chinese population. It may be worth exploring the optimal components for populations with different staple foods by region, economic development status or by food transition progress. Third, temporal trend analysis of dietary patterns at the regional and national level may help public health policymakers estimate potential healthcare needs and plan for proper public health interventions.

## Conclusion

We identified multiple dietary patterns that reflect the diverse dietary patterns among different Chinese populations and the transition of the Chinese diet from a traditional plant-based diet to a westernised and animal food diet. Despite the heterogeneity of the results and the relatively small effects size observed, our results still clearly showed that a ‘healthy Chinese diet’ characterised by whole grains, fresh vegetables, soyabean and products, fish and seafood, mushrooms and fungi, and nuts was associated with better cardiovascular health and reduced risks of metabolism conditions, cognitive impairment and depressive symptoms. Animal-food diet was associated with a higher risk of CVD, diabetes, abdominal obesity and depressive symptoms. Western diet were associated with an increased risk of both general and abdominal obesity and depressive symptoms. The present review provides moderate to high-quality evidence on the associations between identified dietary patterns among the Chinese population and specific health outcomes. Findings on cancer, CVD and diabetes were based on cohort studies. Therefore, the evidence for the negative associations between the healthy Chinese diet, that is, the combination of the traditional WG diet and the Plant-based diet with cancer, CVD and diabetes is strong compared with the moderate evidence of the other health outcomes, including hypertension, cognitive impairment, abdominal obesity and depressive symptoms, which were based on mixed cohort studies and cross-sectional studies. The results of this systematic review and meta-analysis can help inform nutrition and public health policymakers to revert the nutrition transition and reduce disease burdens among Chinese populations.

Future studies with more standardised dietary assessment tools and reporting guidelines – such as a comprehensive summary of daily or annual intake at food group or food item level – would greatly facilitate the comparison and classification of dietary patterns between studies. Additionally, longitudinal studies are needed to strengthen our understanding of the association between Chinese dietary patterns and specific health outcomes, such as particular types of cancer and CVD, and between fatal and non-fatal events. These studies would also help confirm whether the observed associations between metabolic conditions and other health outcomes are consistent over time.

## Supporting information

Hu et al. supplementary material 1 Hu et al. supplementary material

Hu et al. supplementary material 2 Hu et al. supplementary material

## Supplementary material

For supplementary material accompanying this paper visit https://doi.org/10.1017/S1368980024001927
