# Supplementary material for: Identification of Chinese dietary patterns and their relationships with health outcomes: a systematic review and meta-analysis
Source: Public Health Nutr. 2024 Oct 14;27(1):e209. doi: 10.1017/S1368980024001927 (PMC11604330; doi:10.1017/S1368980024001927)
Supplement: Hu et al. supplementary material 2 — Hu et al. supplementary material [file S1368980024001927sup002.docx]

**Table of content**

Supplementary Table 1. PICOS criteria for inclusion and exclusion of studies

Supplementary Table 2. Modified Newcastle-Ottawa scale

Supplementary Figure 1. Count of included studies by region

Supplementary Figure 2. Associations between dietary patterns and cancer in the Chinese population from cohort studies

Supplementary Figure 3. Associations between dietary patterns and cardiovascular disease in the Chinese population from cohort studies

Supplementary Figure 4. Associations between dietary patterns and diabetes in the Chinese population from cohort studies

Supplementary Figure 5. Associations between dietary patterns and hypertension in the Chinese population

Supplementary Figure 6. Associations between dietary patterns and general obesity in the Chinese population

Supplementary Figure 7. Associations between dietary patterns and abdominal obesity in the Chinese population

Supplementary Figure 8. Associations between dietary patterns and cognitive impairment in the Chinese population

Supplementary Figure 9. Associations between dietary patterns and depressive symptoms in the Chinese population

Supplementary Figure 10. Sensitivity analysis: impact of dietary assessment methods on associations between dietary patterns and health outcomes.

Supplementary Figure 11. Sensitivity analysis: impact of study design on associations between dietary patterns and diabetes

Supplementary Figure 12. Sensitivity analysis: impact of participants’ age group on associations between dietary patterns and health outcomes.

Supplementary Figure 13. Sensitivity analysis: associations between Plant-based diet, Traditional whole-grain diet, the combined “healthy Chinese diet” and components of metabolic symptoms in the Chinese population

Supplementary Table 1. PICOS criteria for inclusion and exclusion of studies

| Criteria | Description |
| --- | --- |
| Population | Chinese population living in mainland China, Hong Kong Special Administrative Region (SAR), Macao SAR, Taiwan, and Singapore, and Chinese immigrants living in other countries |
| Intervention | Participants adhere to dietary pattern (with the highest dietary pattern score) in studies with principle component analysis, factor analysis, or reduced rank regression;  Participants with dietary pattern of interest in cluster analysis. |
| Comparison | Participants not adhere to dietary pattern (with the lowest dietary pattern score) in studies with principle component analysis, factor analysis, or reduced rank regression;  Participants with another dietary pattern in cluster analysis. |
| Outcome | Cardiovascular disease from cohort studies, including fatal and nonfatal events: stroke, acute myocardial infarction, and coronary heart disease  Cancer from cohort studies, including fatal and nonfatal events.  Metabolic outcomes from all study designs, including type 2 diabetes, hypertension, general obesity (defined using body mass index), abdominal obesity (defined using waist circumstance), and lipid disorders (including dyslipidemia, hypercholesterolemia, hypertriglyceridemia, and low high-density lipoprotein).  Gestational diabetes  Cognitive impairment  Depressive symptoms |
| Study design | Cross-sectional studies, case-control studies, cohort studies |

Supplementary Table 2. Modified Newcastle-Ottawa scale

| Cohort studies:  1 Is the cohort representative of the average population in the community?  2 Was the dietary intake assessed with a valid tool?  3 Was the dietary pattern derivation methods described in detail?  4 Was the identified dietary patterns described in detail?  5 Demonstration that the outcome of interest was not present at the start of the study?  6 Comparability of cohorts on the basis of the design or analysis: study controls for age and sex?  7 Comparability of cohorts on the basis of the design or analysis: study additionally controls for other factors?  8 Was the follow-up long enough for outcomes to occur?  9 Was the follow-up of the cohorts adequate? |
| --- |
| Case–control and cross-sectional studies:  1 Is the case definition adequate?  2 Were the controls drawn from the same community as the cases?  3 Were the controls defined as having no history of the disease?  4 Comparability of cohorts on the basis of the design or analysis: study controls for age?  5 Comparability of cohorts on the basis of the design or analysis: study additionally controls for other factors?  6 Was the dietary intake assessed with the same valid tool for both cases and controls?  7 Was the dietary pattern derivation methods described in detail?  8 Was the identified dietary patterns described in detail?  9 Was the non-response rate the same for both cases and controls? |


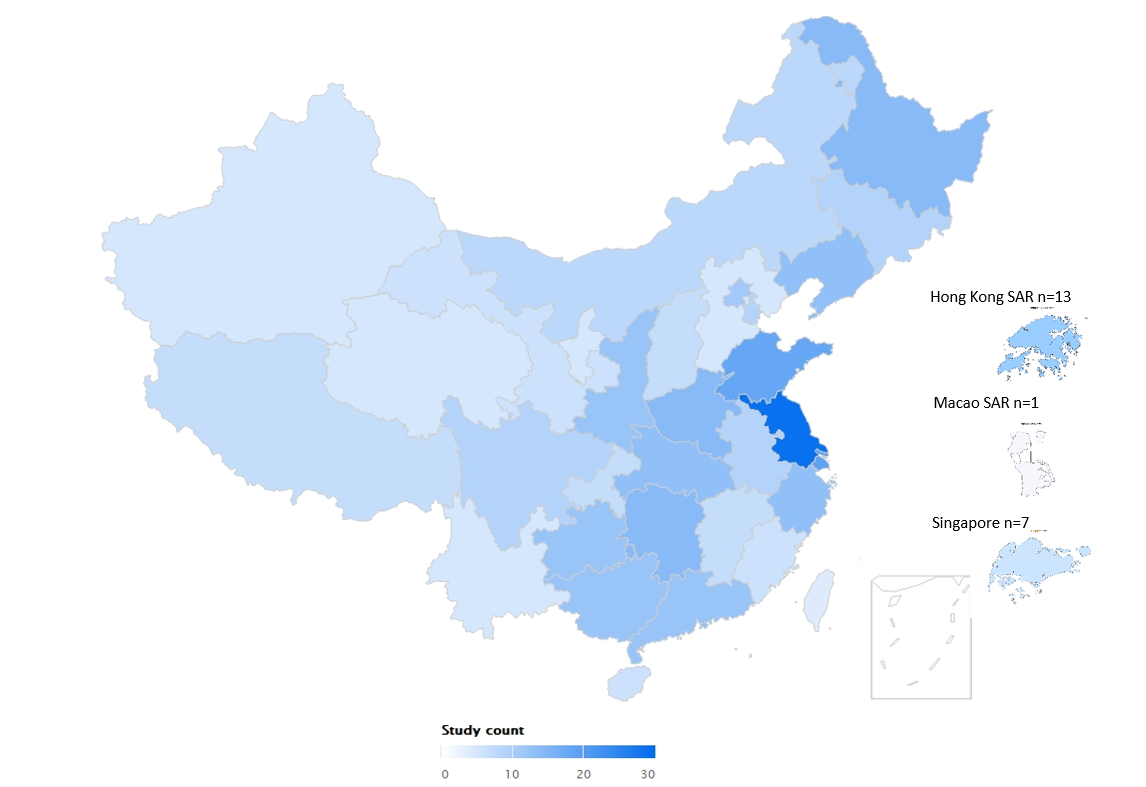


Supplementary Figure 1. Count of included studies by region


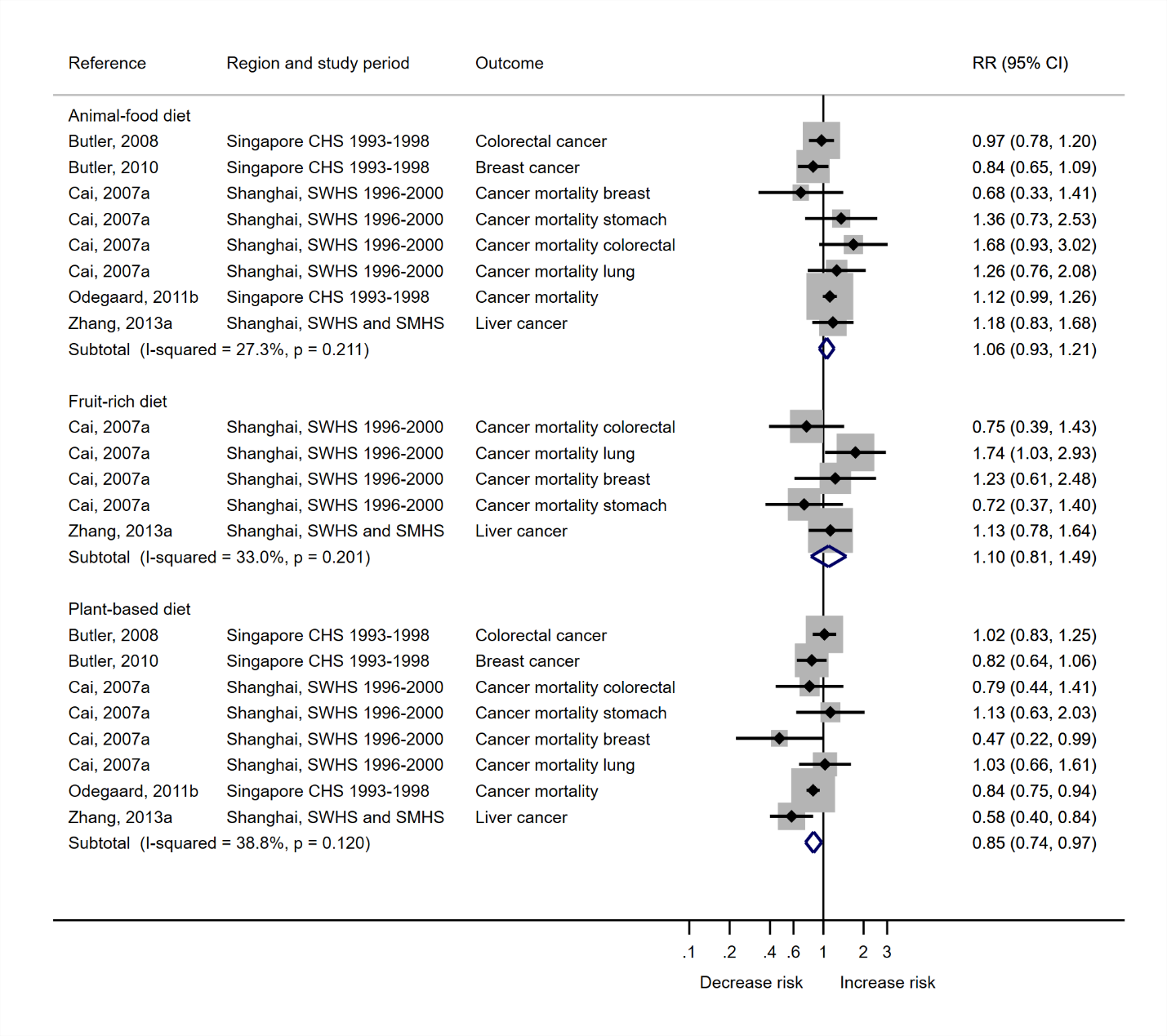


Supplementary Figure 2. Associations between dietary patterns and cancer in the Chinese population from cohort studies


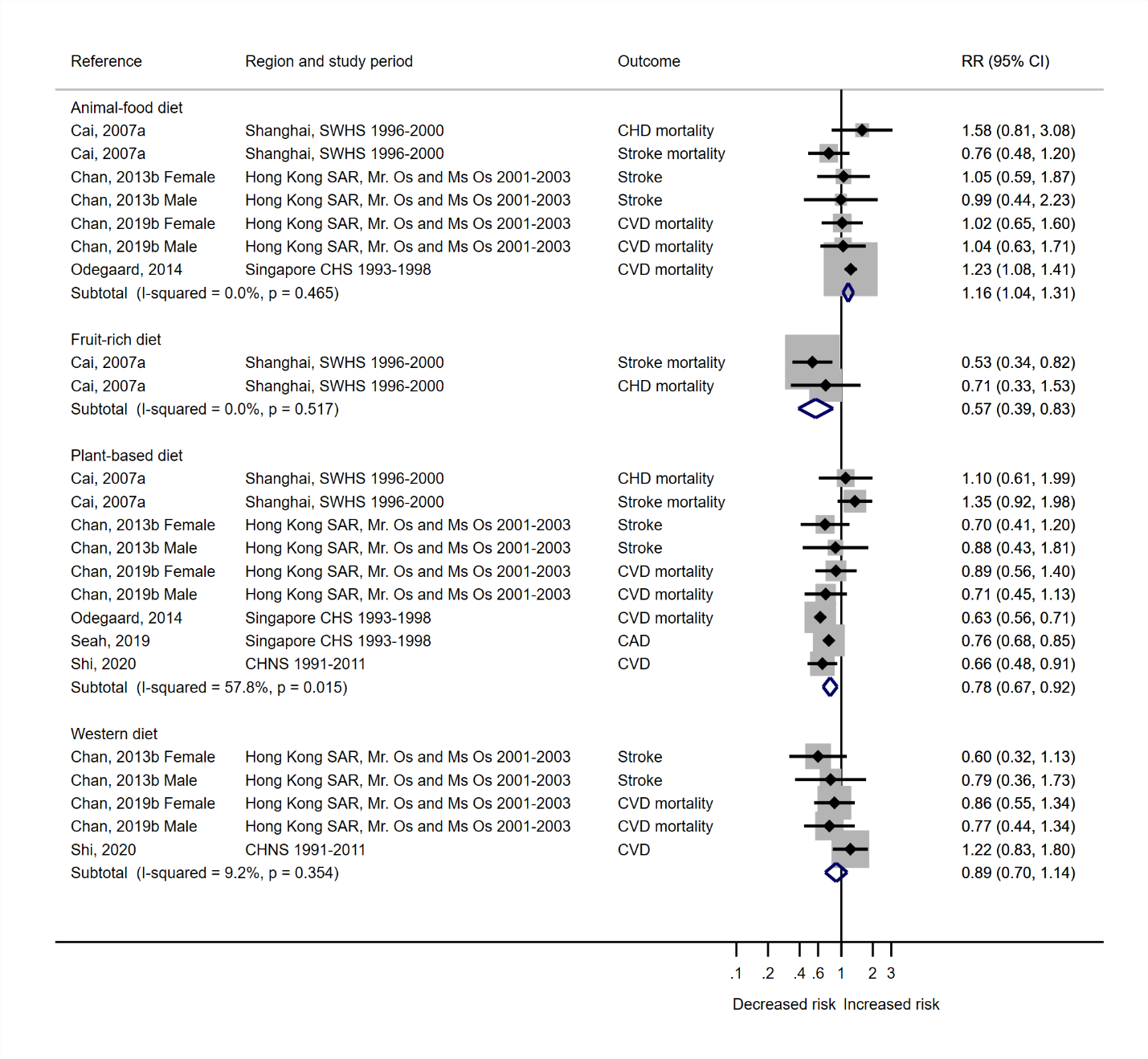


Supplementary Figure 3. Associations between dietary patterns and cardiovascular disease in the Chinese population from cohort studies


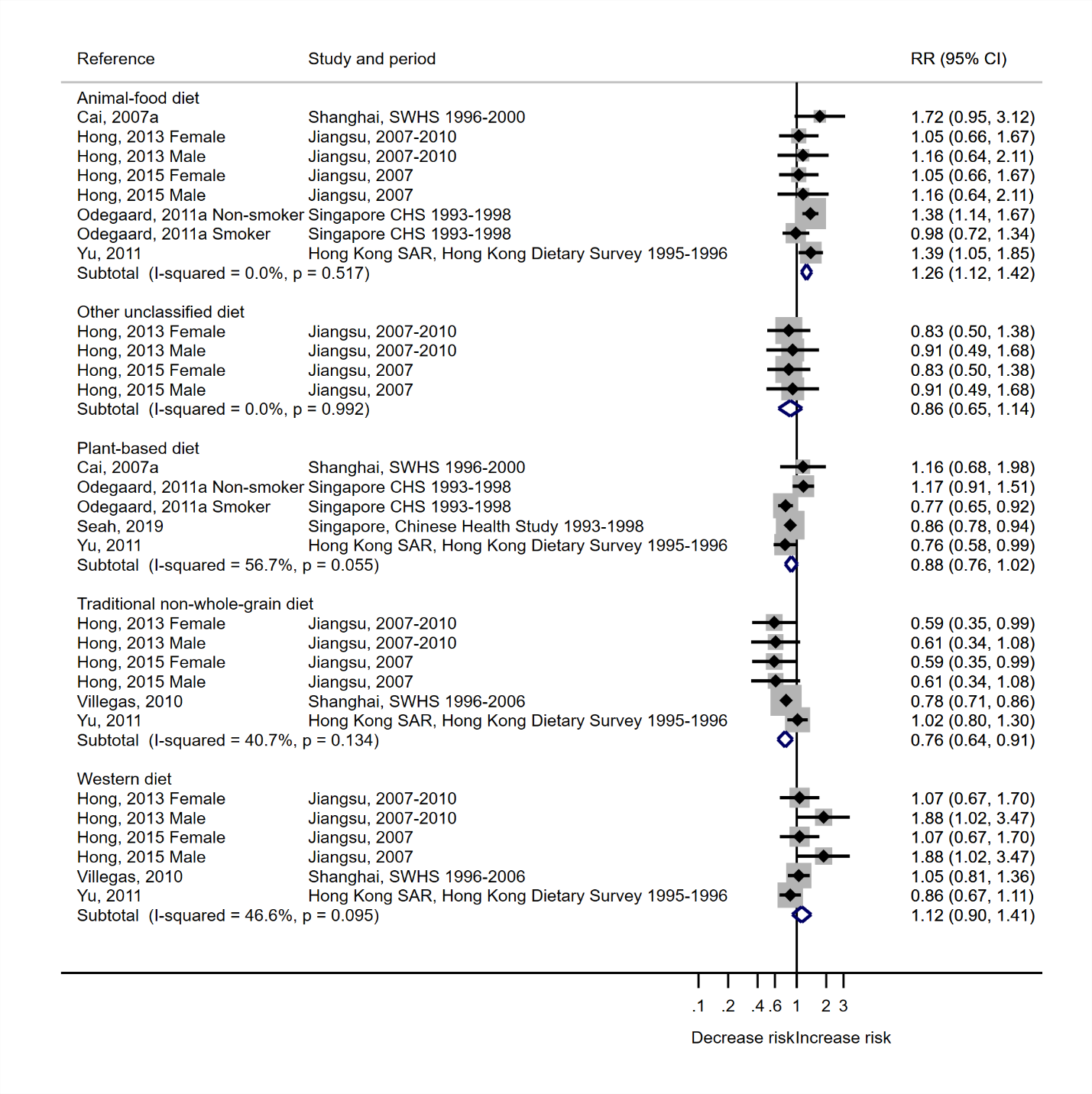


Supplementary Figure 4. Associations between dietary patterns and diabetes in the Chinese population from cohort studies


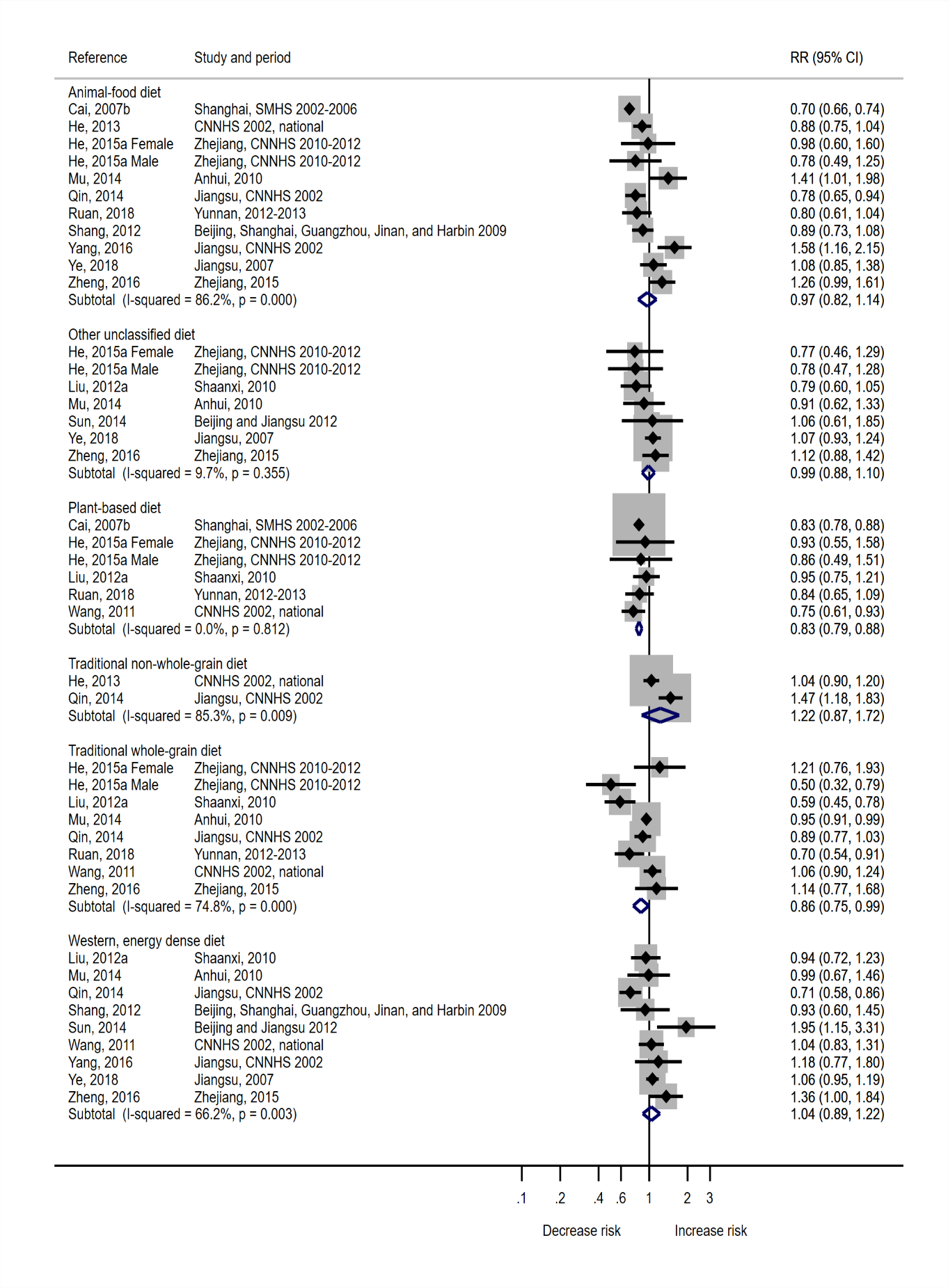


Supplementary Figure 5. Associations between dietary patterns and hypertension in the Chinese population


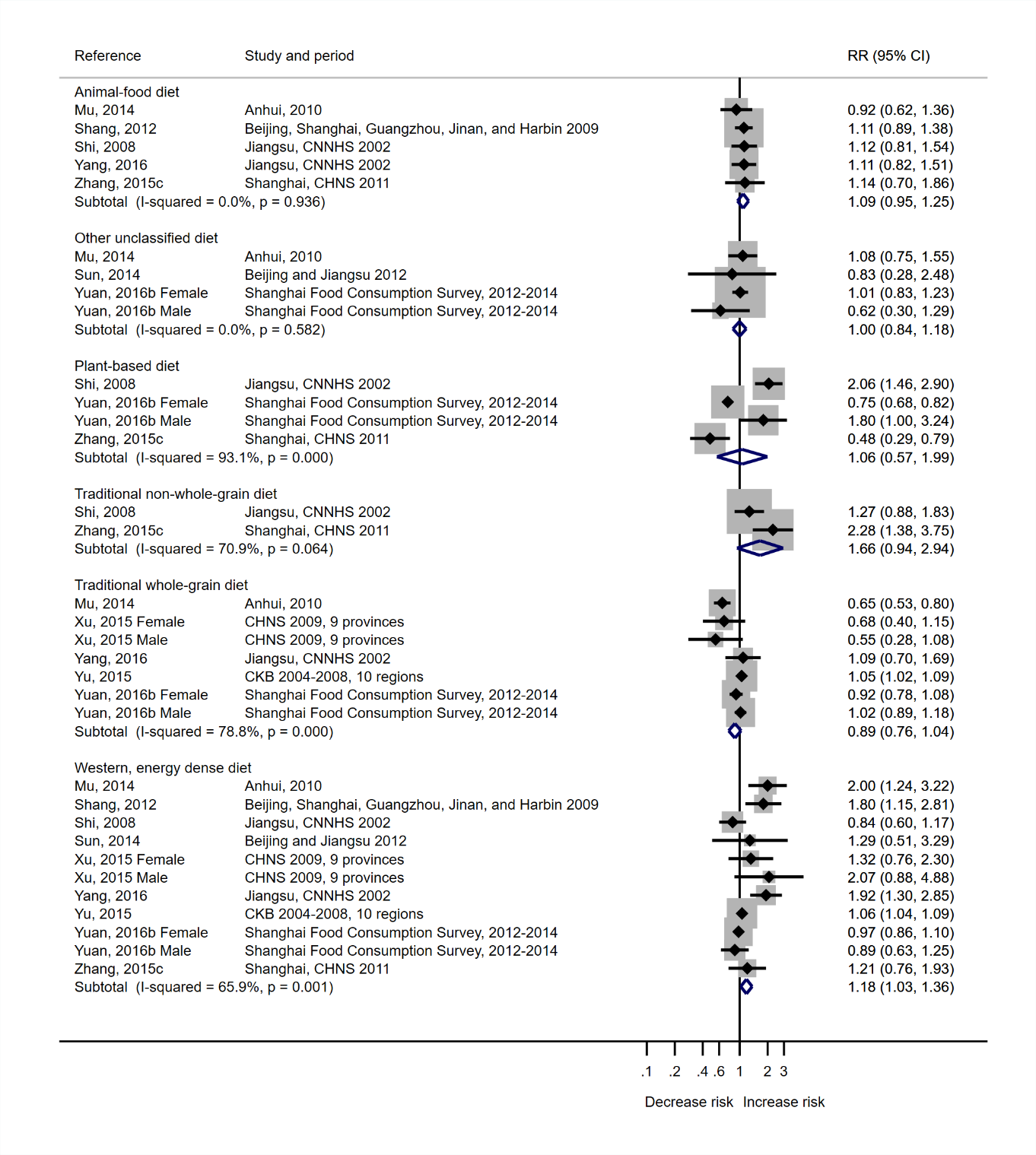


Supplementary Figure 6. Associations between dietary patterns and general obesity in the Chinese population


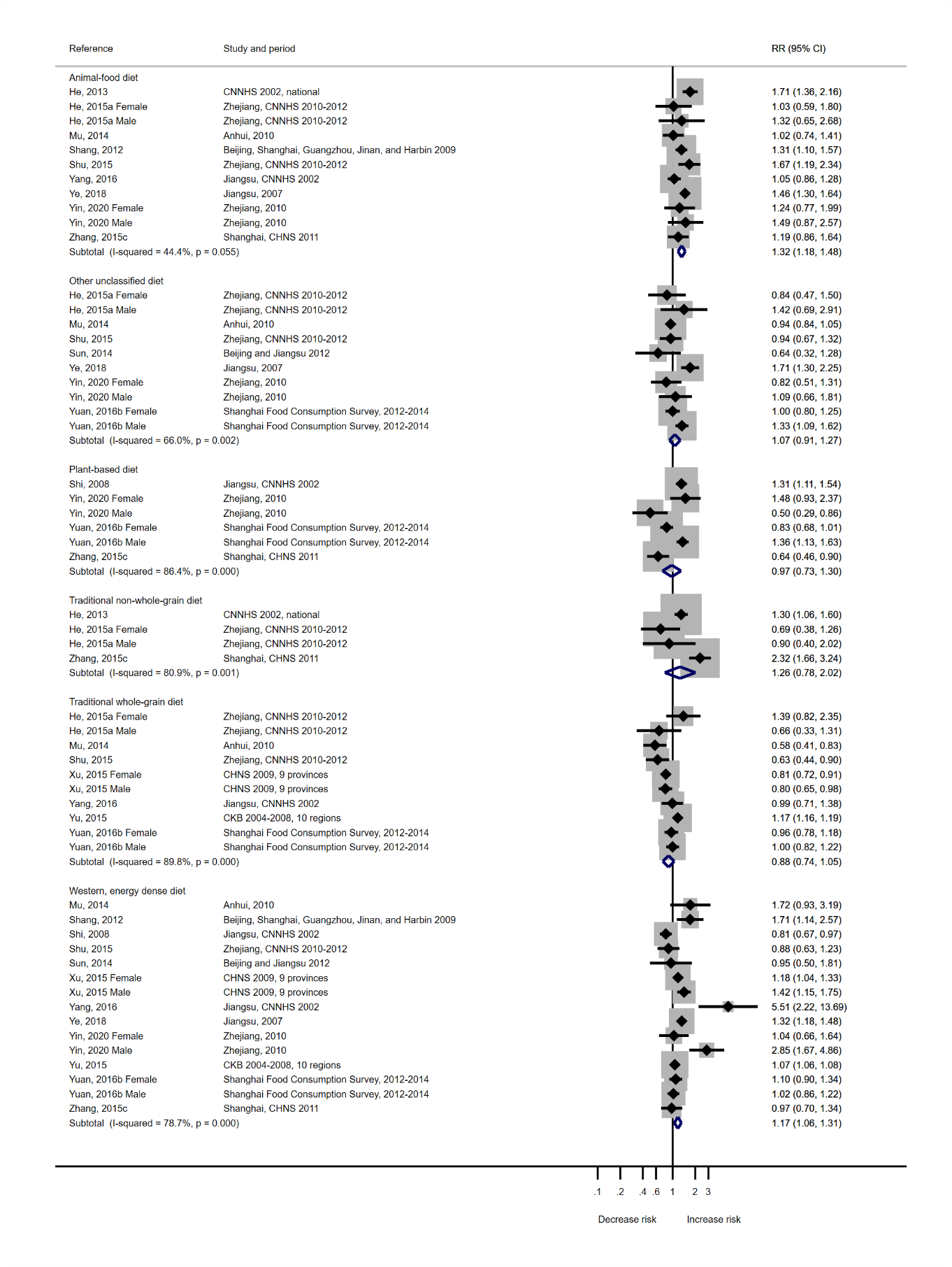


Supplementary Figure 7. Associations between dietary patterns and abdominal obesity in the Chinese population


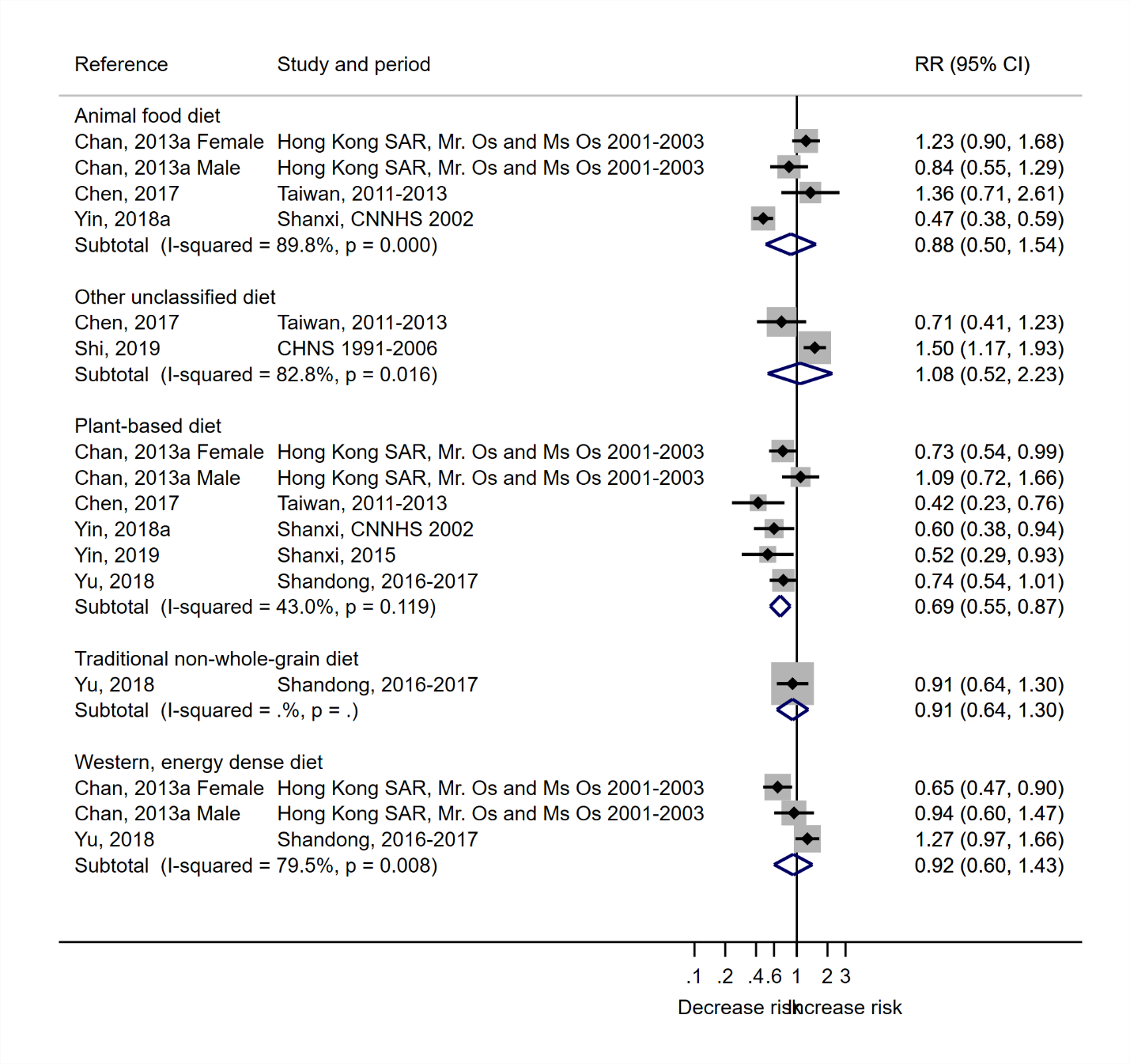


Supplementary Figure 8. Associations between dietary patterns and cognitive impairment in the Chinese population


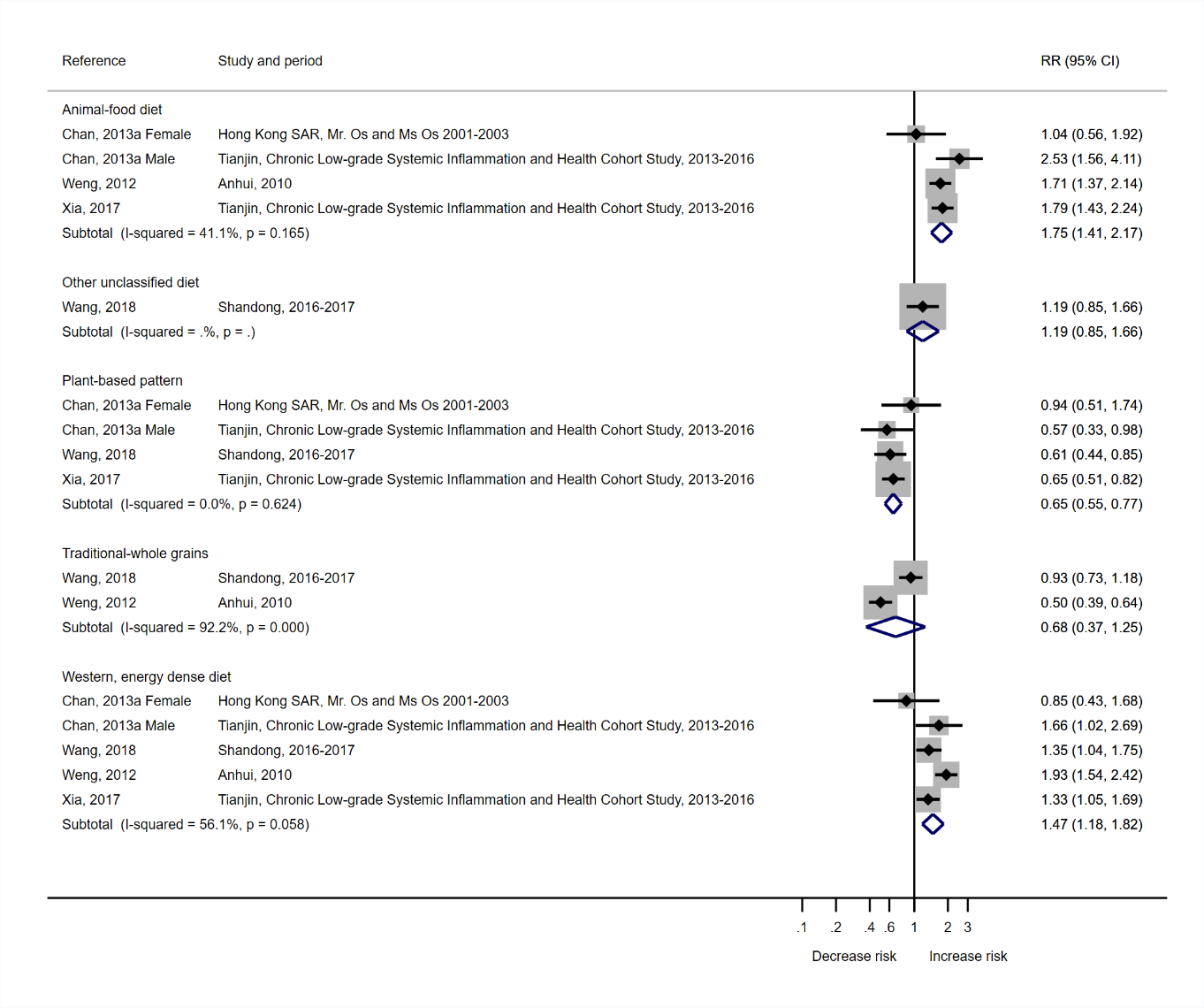


Supplementary Figure 9. Associations between dietary patterns and depressive symptoms in the Chinese population


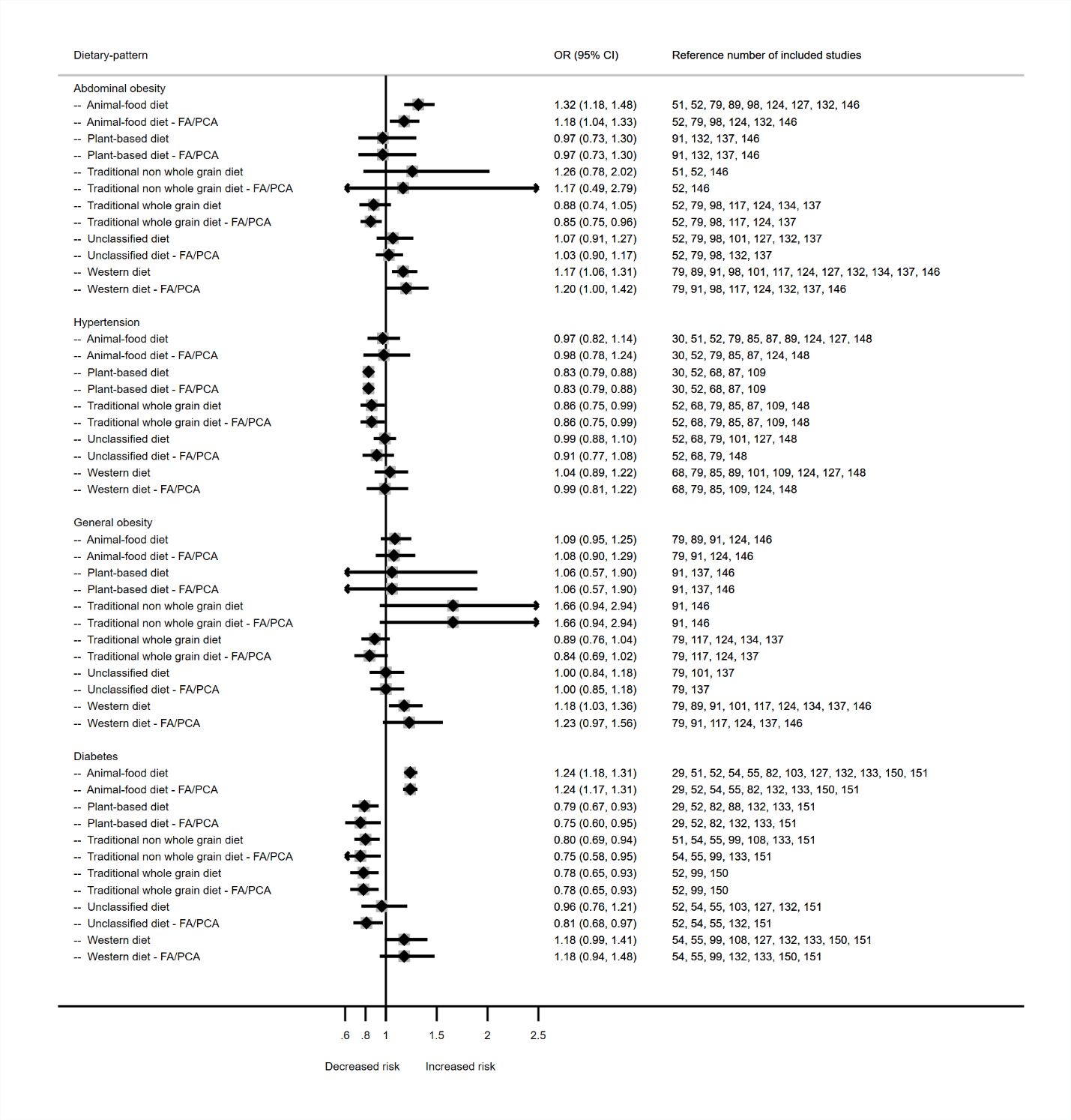


Supplementary Figure 10. Sensitivity analysis: impact of dietary assessment methods on associations between dietary patterns and health outcomes


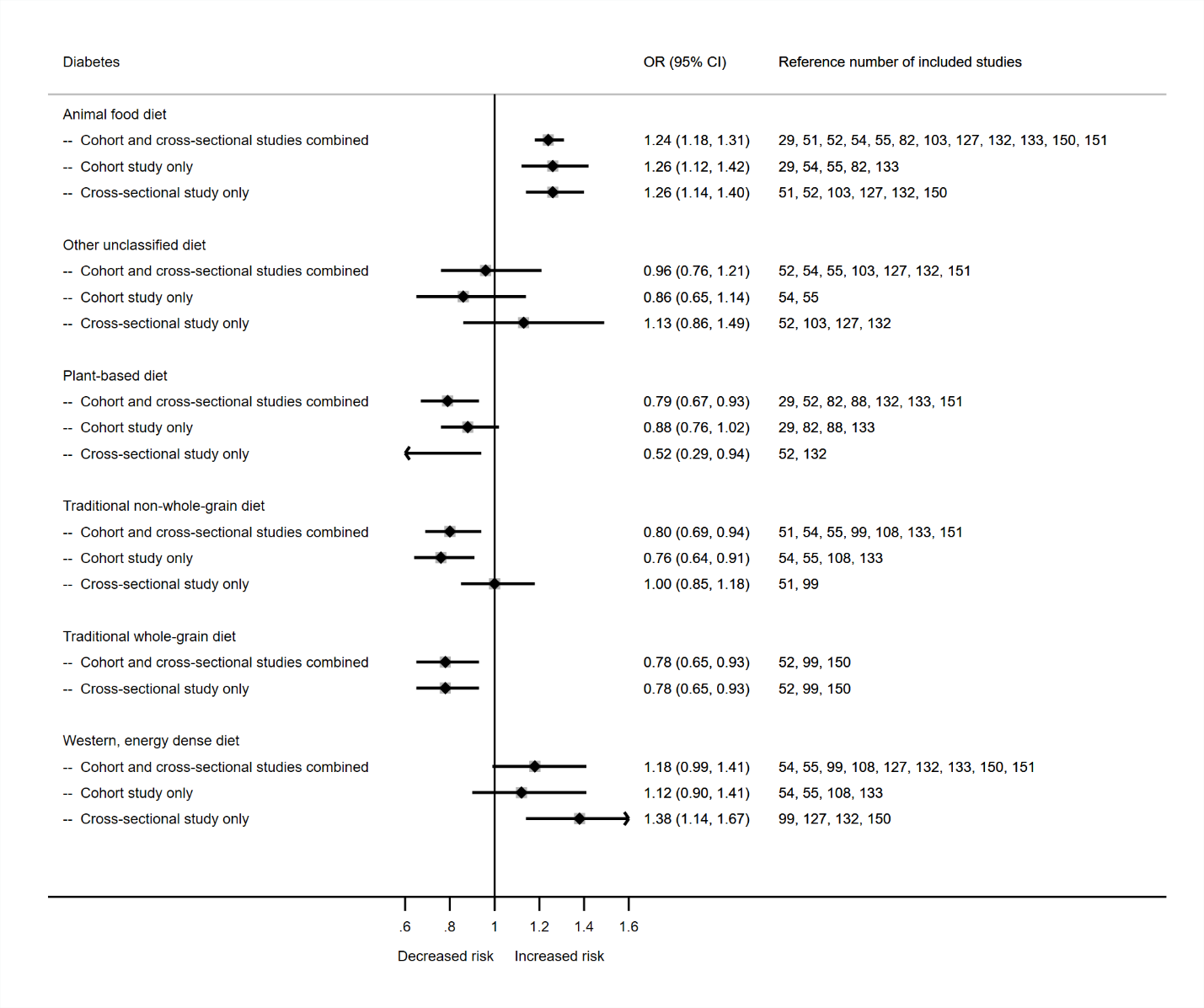


Supplementary Figure 11. Sensitivity analysis: impact of study design on associations between dietary patterns and diabetes


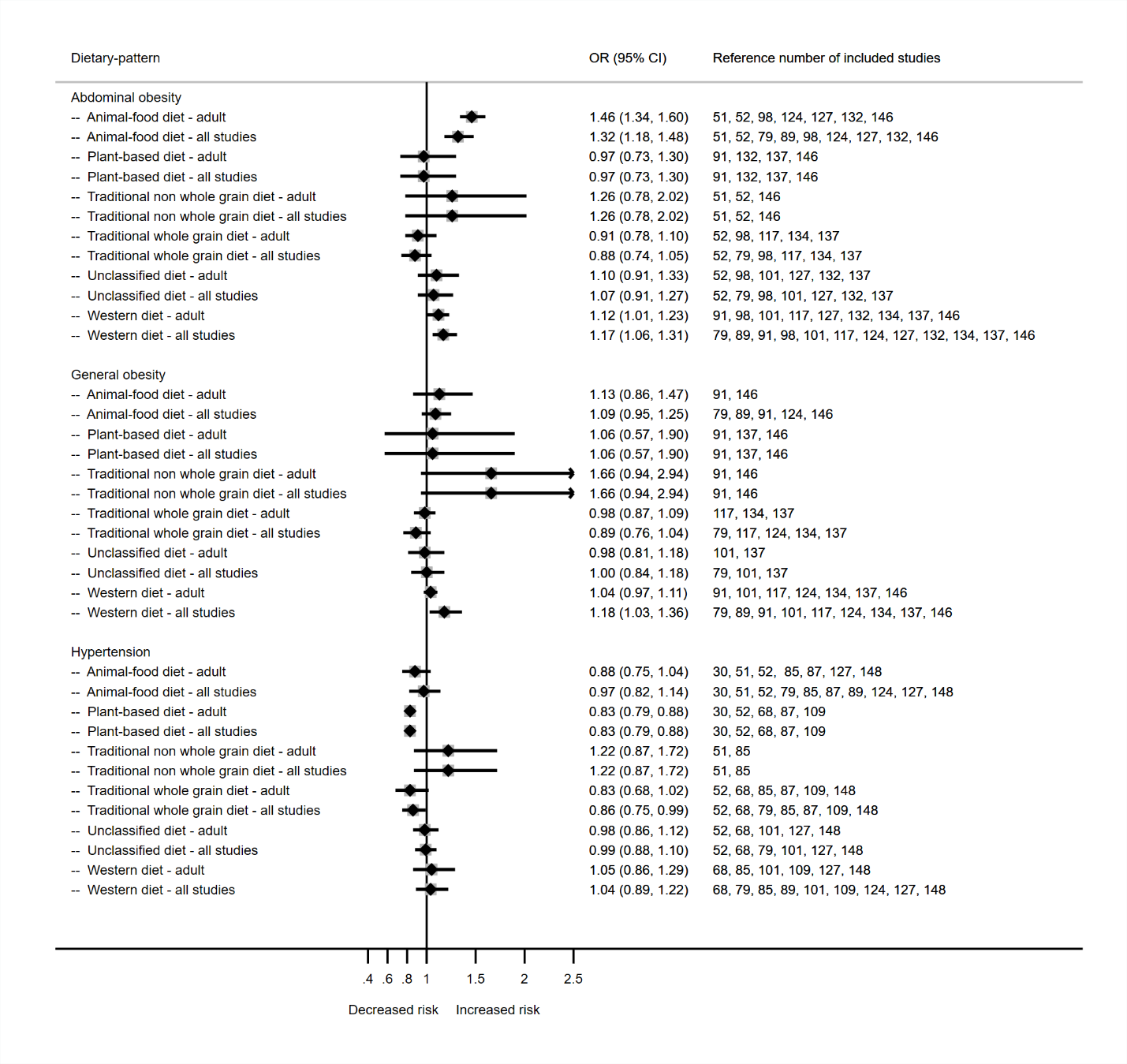


Supplementary Figure 12. Sensitivity analysis: impact of participants’ age group on associations between dietary patterns and health outcomes.


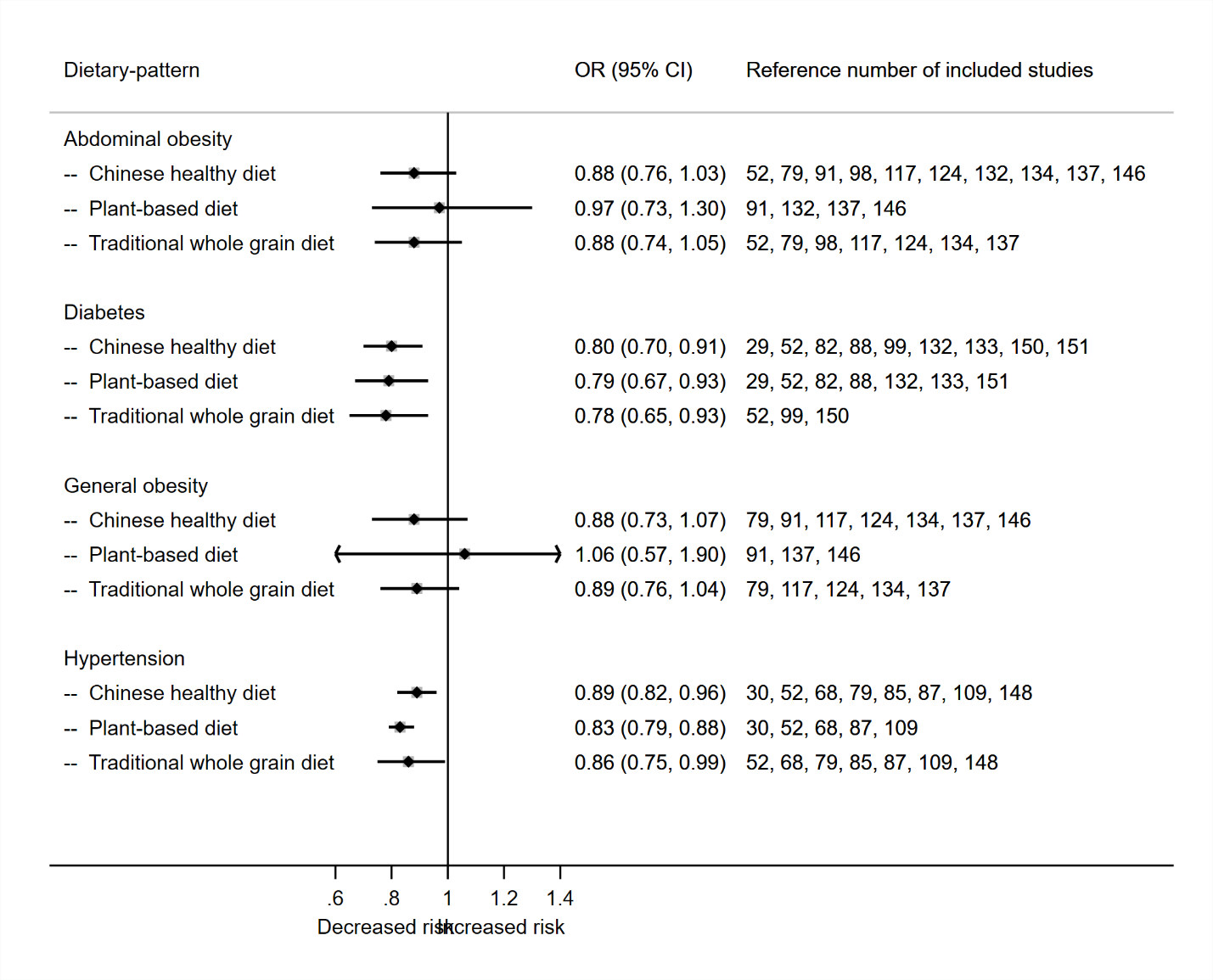


Supplementary Figure 13. Sensitivity analysis: impact of combining plant-based diet and traditional whole grain diet on associations between dietary patterns and health outcomes.
